# Advancements in Mesenchymal Stem Cell-Based Therapy for Enhancing Arteriovenous Fistula Patency

**DOI:** 10.3390/ijms252312719

**Published:** 2024-11-26

**Authors:** Gaurav Baranwal, Haseeb Mukhtar, Jamie Kane, Alaura Lemieux, Sanjay Misra

**Affiliations:** Vascular and Interventional Radiology Translational Laboratory, Department of Radiology, Mayo Clinic, Rochester, MN 55905, USA; baranwal.gaurav@mayo.edu (G.B.); mukhtar.haseeb@mayo.edu (H.M.); kane.jamie@mayo.edu (J.K.); lemieux.alaura@mayo.edu (A.L.)

**Keywords:** arteriovenous fistulas, stenosis and restenosis, mesenchymal stem cells, vascular remodeling

## Abstract

Chronic kidney disease (CKD) affects more than 10% of the world’s population. Hemodialysis, along with peritoneal dialysis and renal transplant, is one of the renal replacement therapies offered to patients with CKD/end-stage renal disease (ESRD). To proceed with hemodialysis, vascular access is required. The two means of long-term access are arteriovenous fistula (AVF) and arteriovenous graft (AVG). Multiple therapies have been created to help the long-term patency of AVFs. These therapies are needed as 40% of AVFs fail within the first year and additional intervention is required. Much of the existing research has focused on biomarkers, immune cells, hypoxia, and cell-based therapies. Regeneration therapy using mesenchymal stem cells seeks to investigate other ways that we can treat AVF failure. Mesenchymal stem cells are harvested as two main types, fetal and adult. Fetal cells are harvested at different times in fetal gestation and from multiple sources, placental blood, Whartons jelly, and amniotic stem cell fluid. Taken together, this review summarizes the different preclinical/clinical studies conducted using different types of MSCs towards vascular regenerative medicine and further highlights its potential to be a suitable alternative approach to enhance AVF patency.

## 1. Introduction

### 1.1. Arteriovenous Fistulas (AVFs) as Preferred Vascular Access for Hemodialysis in Chronic Kidney Disease

Chronic kidney disease (CKD) affects more than 10% of the world’s population [1]. The Kidney Disease: Improving Global Outcomes (KDIGO) organization in 2024 updated the definition of chronic kidney disease (CKD) and provided guidelines for its evaluation and management. CKD is diagnosed when kidney structure or function has been abnormal for more than 3 months with cutoffs of an estimated glomerular filtration rate (eGFR) of less than 60 mL/min per 1.73 m^2^ and an albumin creatinine ratio of 30 mg/g or more [2]. CKD is categorized into five stages based on the eGFR, albumin excretion rate, and cause [3]. The fifth stage, defined by an eGFR of less than 15 mL/min/1.73 m^2^, is also referred to as end-stage renal disease (ESRD). The United States Renal Data Systems (USRDS) reported the latest ESRD prevalence to be 815,142 patients with the main causes being diabetes mellitus, hypertension, and glomerulonephritis [4].

According to NIDDK, between 2001 and 2019, the number of patients with newly registered ESRD increased from 97,856 to 134,837, an increase of 37.8%. Hemodialysis, along with peritoneal dialysis and renal transplant, is one of the renal replacement therapies offered to patients with ESRD [5]. To proceed with hemodialysis, vascular access is required. The two means of long-term access are arteriovenous fistula (AVF) and arteriovenous graft (AVG). The former is preferred due to reduced chances of infection, thrombosis, and stenosis [6,7].

### 1.2. AVFs Failure, Stenosis, and Restenosis

Even though 70% of ESRD patients undergo hemodialysis using an arteriovenous fistula (AVF) as their primary vascular access in the USA, the patency of the AVF declines in ~40% of AVFs due to venous neointimal hyperplasia (VNH) and venous stenosis (VS). Significant stenosis of the AVF is defined as narrowing greater than 50% of the lumen diameter [8]. The first line of treatment for AVF stenosis is percutaneous transluminal angioplasty (PTA). PTA is also used to treat AVFs that have not matured enough to meet the HD requirements within 3 months of creation. AVFs are considered mature if they can be easily punctured with minimal blood leakage during HD, can be used for 3 or more HD sessions a week, and meet the duplex ultrasound criteria as follows: vein diameter > 6 mm, blood flow > 600 mL/min and vein depth < 6 mm from the skin [9,10]. Approximately 200,000 PTA procedures are performed each year to maintain vascular access patency in the US alone [11,12]. Primary patency rates after PTA decline from 61% to 35% at 6 and 24 months, respectively [11]. The recent literature has found that drug-eluting balloon (DEB) angioplasty has significantly better 6- and 12-month patency rates when compared to conventional PTA. The studies include a randomized controlled trial in their meta-analyses which suggests that DEB is a safe and effective treatment compared to PTA. However, there are reports suggestive of an increased risk of death with paclitaxel-coated balloons and stent in the femoral artery of the leg, so further studies are needed to understand the potential associated with DEB [13,14,15]. Considering these facts, there is an immense need to further explore alternative therapies to enhance AVF patency and understand in detail the current therapeutic approaches which might pave the way to finding alternative treatments.

## 2. Current Therapeutic Approaches to Prevent AVF Stenosis

There is a significant amount of work which has been conducted to define and understand various novel therapeutic approaches to treat VS and VNH of AVFs. Most of these studies seek to understand and target the related immune cells, associated signaling molecules, hypoxia, and utilize cell-based therapies. In addition, the route of dispersal has been an important criterion to advance these therapeutic approaches: systemic vs. perivascular.

### 2.1. Targeting Cytokines and Chemokines

Studies have identified clinical readouts that can act as biomarkers in patients. For example, higher systemic inflammatory markers like platelet-to-lymphocyte and neutrophil-to-lymphocyte ratios are risk factors for early AVF restenosis after PTA [16,17]. Targeting inflammatory molecules has shown positive results in preventing the restenosis of AVFs, including the blockade of TLR-4 to attenuate thrombosis, inflammation, and stenosis via regulating VSMCs’ migration and proliferation [18], and the inhibition of β-catenin, which leads to the blocking of the pathways associated with endothelial to myofibroblast trans-differentiation, causing vascular thickening in AVFs [19]. A major inflammatory response during AVF stenosis and restenosis is the infiltration of macrophages and a change in the dynamics of M1-like and M2-like macrophages. Targeting the maturation and migration of macrophages has been reported to have a significant impact on enhancing the patency of AVFs; for example, the blockade of CX3CR1 signaling inhibits the migration of macrophages and thus attenuates fibrosis [20]. Atorvastatin (ATV) treatment reduces macrophage accumulation, leading to reduced venous stenosis and improved AVF patency by positive AVF venous limb outward remodeling and reduced collagen deposition in the vein wall. In the same study, nanoparticle enhanced imaging showed a significant reduction in macrophage accumulation in vivo, which further enhances the patency of AVF [21]. Macrophages, endothelial cells, and vascular smooth muscle cells (VSMCs) all express monocyte chemoattractant protein-1 (MCP-1), which can have a great impact on the migration of monocytes or macrophages, leading to associated inflammatory responses during intimal hyperplasia (IH) and atherogenesis [22,23]. MCP-1 recognizes CC chemokine receptor 2 (CCR2) as its functional receptor and has been reported to actively recruit VSMCs and cause the progression of IH in animal models of chronic arterial injury [24]. The local adventitial delivery of nanoformulated Bindarit, a selective inhibitor of MCP-1, has been shown to significantly prevent VIH progression and restenosis in a murine model of AVF [25]. A systemic review by Wang et al. also summarized other major inflammatory molecules and inflammatory cells as therapeutic targets for individualized AVF stenosis/VNH prevention-associated treatments [26].

### 2.2. Cell-Based Therapies

However, considering the extensive cellular and inflammatory responses during AVFs stenosis and restenosis, cell-based therapies may be a more promising approach to prevent the cascade of signaling changes at the cellular and molecular level. The biology of the vascular endothelium is critical to understand vascular pathology and associated injury. Endothelial cells regulate not only the local boundary for circulation but also modulate the biological functions affecting the physiology of the vessels. The use of endothelial cells for therapeutically healing the vasculature is an emerging field of interest. A tissue graft of gel foam containing aortic endothelial cells isolated from porcine and bovine sources reduced the restenosis index by 54% and 46% [27]. Multiple preclinical reports suggested that grafts containing bone marrow-derived endothelial cells, blood outgrowth endothelial cells, and allogenic and xenogeneic endothelial cells are helpful to prevent neointimal hyperplasia and fibrosis and promote neovascularization. These effects were predominantly induced by increasing matrix metalloprotein (MMP) expression and reducing hypoxia-induced factors (HIFs) [28,29,30]. Furthermore, a multicenter phase I/II trial for evaluating the safety and efficacy of Vascugel sponges, an allogeneic endothelial cell implant, was conducted after the creation of arteriovenous access. This study reported that the allogenic transplantation of endothelial cells was safe and effectively enhanced the patency of AVFs/AVGs. This study showed that targeted local therapy with perivascular and allogenic endothelial cells is a safe and novel therapeutic approach based on phase I (followed for 30 days) and phase II (24-week follow-up) study data [31]. Recent developments have included utilizing biotinylated endothelial cells to avidin-functionalized stents in rats. This methodology helps to reduce endothelial cell destruction after delivery and stenotic responses. However, it is unknown if this methodology may apply to AVF treatments as very few studies have been performed with vascular access [32]. Similarly, the transplantation of platelet-derived growth factor receptor (PDGFR)-β endothelial progenitor cells (EPCs) accelerates reendothelialization, with the enhanced apoptosis of VSMCs 14 days after injury. This is one of the first studies using genetically modified EPCs to reduce neointima formation in a mouse carotid artery injury model both in vitro and ex vivo [33]. Endothelial-based therapies such as these may not be effective or appropriate in all patients, and, to date, there have been no therapies that have progressed to approval and clinical application. Allogeneic therapies like these may induce an inflammatory rejection-like response in a tissue and or AVF, where it may be most damaging. Additionally, ex vivo culture and preparation of a sufficient quantity and quality of endothelial cells to be used in these applications present significant challenges, which remain the subject of ongoing studies [25].

Thus, alternative cell sources are required to enhance cell-based therapies. Recent work from our group has shown promising results from the application of mesenchymal stem cells (MSCs) to improve the patency of AVFs in preclinical and clinical scenarios [34].

## 3. Application of Mesenchymal Stem Cells for Vascular Regeneration

### 3.1. Mesenchymal Stem Cells as an Attractive Candidate for Cell-Based Therapies to Improve AVF Patency

Mesenchymal stem cells (MSCs) were first discovered by Friedenstein et al. [35] in the 1960s and termed colony-forming fibroblast-like cells in vitro. Since then, many developments have been made to characterize and define the markers and potency of these cells to reveal their therapeutic potential. Based on the ISCT (International Society of Cellular Therapy) guidelines, in 2006, these multipotent MSCs were defined by their ability to adhere to a plastic surface, to differentiate minimally into three lineages of osteogenic, chondrogenic, and adipogenic cells, to express specific surface markers: >95% (CD73, CD90, CD105), and their lack of immune cell markers like CD45, CD34, CD14, CD11b, CD79a, and CD19 or major histocompatibility complex (MHC) class II. There are other markers based on MSC origin, including CD9, CD10, CD13, CD29, CD44, CD49, CD51, CD54 (ICAM-1), CD117 (c-kit), CD146 (MCAM), CD166 (ALCAM), and Stro-1. Although a wide range of positive markers describing MSCs have been identified, no single marker has been indicated as specific for MSCs [36,37,38].

**Figure 1:**
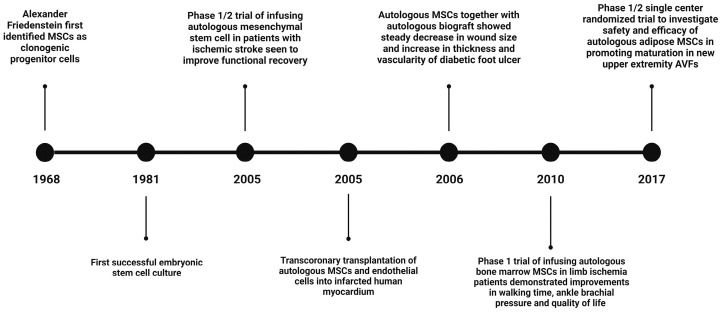
Timeline of MSCs to make their way towards their application in AVF (figure made using Biorender.com).

There has been a plethora of studies from the discovery of MSCs to their applications in clinical trials of various diseases. Figure 1 illustrates the timeline and important reports emphasizing MSC applications in vascular disease, leading towards its therapeutic application in AVF surgeries [35,39,40,41,42,43,44,45].

### 3.2. Source of Stem Cells

This section focuses on MSCs as used in AVF regenerative medicine broadly divided into two main categories based on tissue origin: fetal and adult.

#### 3.2.1. FETAL

Umbilical cord MSCs (UC-MSCs) are one of the main sources of fetal MSCs [46]. UC-MSCs are mainly obtained from Wharton’s jelly, the gelatinous mucous substance surrounding the vessels of the umbilical cord. These UC-MSCs have shown promise in treating a range of diseases including kidney disease [47], respiratory distress [48], thrombocytopenia [49], and skin rejuvenation [50].

US-MSCs are effective immunomodulators, which are important in controlling AVF responses. In a lung fibrosis context, US-MSCs inhibited the expression of transforming growth factor-beta, interferon-gamma, and tumor necrosis factor [48]. Additionally, US-MSCs were able to efficiently change the cell matrix, increasing the expression of matrix metalloproteinases and reducing related endogenous inhibitors.

Specifically, to the AVF context, UC-MSCs have shown promise in preventing neointimal hyperplasia in AVFs by stabilizing hypoxia inducible factor 1α (HIF-1α) in a rabbit model. HIF-1α is a transcriptional factor which plays an important role during cellular responses to hypoxia. If the AVF is insufficiently oxygenated, then intimal hyperplasia may be induced secondary to HIF-1α activation, which is a critical complication associated with AVF failure. Increased HIF-1α expression has been previously identified in the endothelium of maturing AVFs and is correlated to maturation. This may be effective as a biomarker to identify an AVF at risk, but the authors also sought to understand if it could be modulated by UC-MSCs treatment. A significant reduction in HIF-1α was found in the groups treated with UC-MSCs, which in turn promoted angiogenesis and prevented the growth and migration of smooth muscle cells from the tunica media into the tunica intima [51]. Additionally, these effects were found using both in situ injection and intravenous administration.

In coronary artery disease as well as AVF remodeling, rapid reendothelization is a key step to prevent IH-induced restenosis. Kim et al. studied carotid artery injury in a rabbit model, where a ballon-injured coated artery treated with UC-MSCs plus fibrin matrix showed a rapid reendothelization compared to only fibrin matrix and hence resulted in a reduction in IH. The injected UC-MSCs were delivered to the artery in question and were persistent in place for four weeks. This phenotype was accompanied by increases in angiogenic genes such as vascular endothelial growth factor-1, angiopoietin-1, and angio-associated migratory cell protein [52]. In addition to endothelial changes, the authors also describe changes in the immunology of the artery. MSC injection resulted in reduced macrophage accumulation at the site of injury, possibly modulated by the release of interleukin-10. In a separate study treating foot ulcers by angioplasty, UC-MSCs were endovascularly perfused concomitant to the procedure. This intervention resulted in improvements in skin temperature, the ankle-brachial pressure index, transcutaneous oxygen tension, and claudication distance. As a result, the formation of VNH was reduced [53].

Relatedly, MSCs derived from fresh blood in the umbilical cord vein (UCB-MSCs) after childbirth contain a rich source of EPCs. The advantages of this source are its simplicity, safety, and lack of pain. EPCs derived from cord blood have demonstrated promise in vascular repair when compared to adult peripheral blood EPCs in animal models in terms of developing a normal functioning blood vessel which last for more than 4 months (cord blood EPCs) vs. 3 weeks (adult EPCs) when implanted in the cranial window of a severe combined immunodeficient (SCID) mice. Another study showed enhanced vascularization when a mixture of EPCs human umbilical cord blood or from adult peripheral blood and human saphenous vein smooth muscle cells (HSVSMCs) was implanted on the back of a 6-week-old male athymic nu/nu mouse subcutaneously [54,55,56]. Using a rabbit diabetic vascular lesion model, Ding et al. showed that the transplantation of UCB-MSCs combined with PTA reduced stenosis by the inhibition of the migration and proliferation of vascular smooth muscle cells. These effects were likely due to changes in the JAK/STAT3 pathway [57]. Furthermore, in a study investigating ischemic bowel disease, UCB-MSCs infused through the superior and/or inferior mesenteric artery, and the growth hormone used to promote stem cell differentiation after the operation was able to reduce ischemia and increase luminal diameter. The patients from this trial had improved white blood cell counts, improved neutrophil counts, and improved platelets. These changes were likely secondary to the secretion of growth factors by UCB-MSCs. These factors are hepatocyte growth factor, IGF, VEGF, bFGF, and the anti-inflammatory cytokine IL-10 [58], which promote angiogenesis and block inflammatory reactions. It is therefore tempting to hypothesize that this utility would extend into the AVF. Finally, UCB-MSCs may be beneficial in terms of modulating nitric oxide availability and aiding in the dilation of the vascular bed. UCB-MSCs effectively reduced nitric oxide synthase inhibitors and promoted nitric oxide level accumulation to accelerate wound healing in this context, but they also more broadly cause vasodilation [59].

Human amniotic fluid stem cells (AFSCs) are another significant fetal source derived stem cells. A mouse model of hind limb ischemia demonstrated that AFSC-conditioned media administrated by intramuscular weekly injections (topically applied to thigh muscles) evoked a strong proangiogenic response and promoted the cryoprotection, differentiation, and chemoattraction of endothelial cells mainly driven by proangiogenic factors MCP-1, VEGF, and IL-8 presence [60]. The final source of fetal MSCs is placenta-derived mesenchymal stem cells (PL-MSCs). These cells are acquired from the mid gestation/full-term placenta and have shown multilineage differentiation potential [61]. *Wnt* signaling helps in positive vascular remodeling. PL-MSCs have been shown to express CD349/frizzled-9, a receptor for Wnt signaling. Tran et al. showed that two different populations of PL-MSCs, CD349+, and CD349− had a similar pattern of cell surface markers, but CD349− cells have significantly increased blood flow by reendothelization in a bone-fractured mouse model [62]. In another study, ovarian function was significantly improved by the intravenous transplantation of PL-MSCs in an ovariectomized rat model via vascular remodeling by *Wnt* signaling activation mainly driven by hepatocyte growth factor (HGF) secretion by PL-MSCs [63]. Another in vitro study showed conditioned medium (CM) derived from PL-MSC/tissue contained proangiogenic factors CXCL-5, GRO, IL-6, IL-8, and MCP-1 and promoted in vitro angiogenesis in HUVEC [64]. Collectively, the current literature strongly suggests a role for PL-MSCs in positive vascular remodeling mainly by modulating *Wnt* signaling, secreting angiogenic factors, the upregulation of C-reactive proteins (CRP), as well as significantly reducing M1/M2 macrophage inflammations in different models of vascular injuries/limb ischemia/cardiac repair [65,66,67]. However, there are important considerations to be considered for the route of administration, site of distribution, and number of cells [68].

#### 3.2.2. ADULT

A variety of adult tissue origin MSCs have also been studied for vascular regenerative medicine. The most extensively studied in the field of AVF is human adipose tissue-derived MSCs (AMSCs). Upon adventitial transplantation to the AVF, AMSCs have been shown to decrease MCP-1 expression in the vessel wall. This was accompanied by an increase in lumen vessel area and a decrease in the mitotic index, suggestive of decreased proliferation and decreased venous neointimal hyperplasia in murine AVF models. The approach proved practical as the prolonged retention of MSCs was demonstrated at the adventitia as evidenced by serial PET images [69]. Vessels treated with AMSCs in these models have also shown decreased vascular inflammation with the reduced expression of IL-1β and TNF-α and an increased M2/M1 macrophage ratio. Fibrosis was decreased and vascular remodeling was improved with a better response to PTA in AMSC-treated vessels [34]. Bone marrow (BM-MSCs) is another heavily studied MSCs source for AVFs. Bioresorbable perivascular scaffolds loaded with BM-MSCs placed on AVFs demonstrated decreased neointimal hyperplasia and infiltration of inflammatory cells, smooth muscle cells, and fibroblasts. The lumen diameter, wall-to-lumen ratio, and flow rate of the treated vessel increased as seen on an ultrasound, and the uptake on 18F-FDG-PET in the tissue decreased, thereby signifying reduced inflammation. This altogether promoted AVF maturation and vessel patency [70]. In another study, a transient ischemia rat model of middle cerebral artery occlusion, the intravenous administration of BM-MSCs, especially when preconditioned to hypoxia, was shown to more avidly undergo endothelial cell differentiation, upregulate pro-survival and pro-regenerative genes, and downregulate inflammatory genes [71]. Another study showed how BM-MSC transplantation through the femoral vein attenuates cardiac remodeling of diabetic cardiomyopathy in rat models. BM-MSCs led to increased myocardial arteriolar density and decreased collagen volume in the myocardium, thereby improving cardiac function [72]. BM-MSCs may also decrease M1 macrophage population and inhibit the secretion of proinflammatory factors by these cells when injected through the tail vein after a rotator cuff reconstruction in rats [73].

There are some other MSC populations that have been studied for vascular regenerative medicine and could potentially be used in the field of AVF treatment. One of these investigated for endothelial repair is gingival MSCs (GMSCs). Similarly to AMSCs, GMSCs also expressed endothelial markers when co-cultured with endothelial cells (EC); however, the extent to which the proliferation and migration of smooth muscle cells (SMCs) were inhibited by GMSCs was lower than that seen with AMSCs [74]. High concentrations of TNF-α also decrease the endothelial healing and trophic capabilities of GMSCs [75]. Another source that carries great promise is muscle-derived mesenchymal stem cells (MMSCs). The ease of collection is a benefit of MMSCs, as well as their multipotency, as demonstrated by trilineage differentiation into osteoblasts, chondroblasts, and adipoblasts. They have also shown immunomodulation by inhibiting myeloperoxidase in a dose-dependent fashion and reducing reactive oxygen species produced by neutrophils [76]. Dental pulp stromal cells (DPSCs) have also been studied in the domain of regenerative medicine. They have also demonstrated ease of collection and have been attributed as MSC-like cells in virtue of their plastic adherence, surface marker expression, and trilineage differentiation [77]. DPSCs have shown desirable paracrine effects on endothelial cell migration and angiogenesis. However, their potency and extravesicular vesicle production has been observed to be less than that of BM-MSCs [78]. Other sources of mesenchymal stem cells that have not been sufficiently studied in the field of vascular medicine but have regenerative capabilities include synovium and synovial fluid-derived MSCs and endometrial-derived MSCs [79,80].

## 4. Mechanistic Pathways Modulated by Mesenchymal Stem Cells Driving Positive Vascular Remodeling

There are several factors which make MSCs an important therapeutic tool in the treatment of different diseases, such as secreting paracrine factors, immune modulation, and signaling molecules [81]. Notch signaling is one of the important signaling pathways which MSCs possibly modulate to result in positive vascular remodeling. Notch signaling pathways affect how vascular remodeling occurs; this is due to its natural ability to signal efficiently with vascular smooth muscle cells (VSMCs). This signaling to the VSMCs allows the cells to proliferate at a faster pace than in a normal system. The interplay of Notch activation and bone marrow-derived fibroblast-specific protein-1 (FSP1)^+^ cells has been reported as one of the key mechanisms leading to intimal hyperplasia (IH) and AVF failure [82]. Notch signaling-modified MSCs have been reported to induce arteriogenesis, which improved left ventricular function in a rat myocardial infract model [83]. In another study, the same Notch signaling-modified MSCs showed improved tissue perfusion in a rat model of hindlimb ischemia [84].

The other important signaling pathway in the AVF is heme oxygenase 1/peroxisome proliferator-activated receptor gamma (HO-1/PPAR-*γ*). AVF placement significantly elevates HO-1 and PPAR *γ* prior to VS formation in a mouse model [85]. Our lab has recently investigated MSC mediation of AVF maturation to increase the patency of the AVF due to its immunomodulatory properties, helping to promote an anti-inflammatory microenvironment in mouse and porcine models (unpublished data).

**Figure 2:**
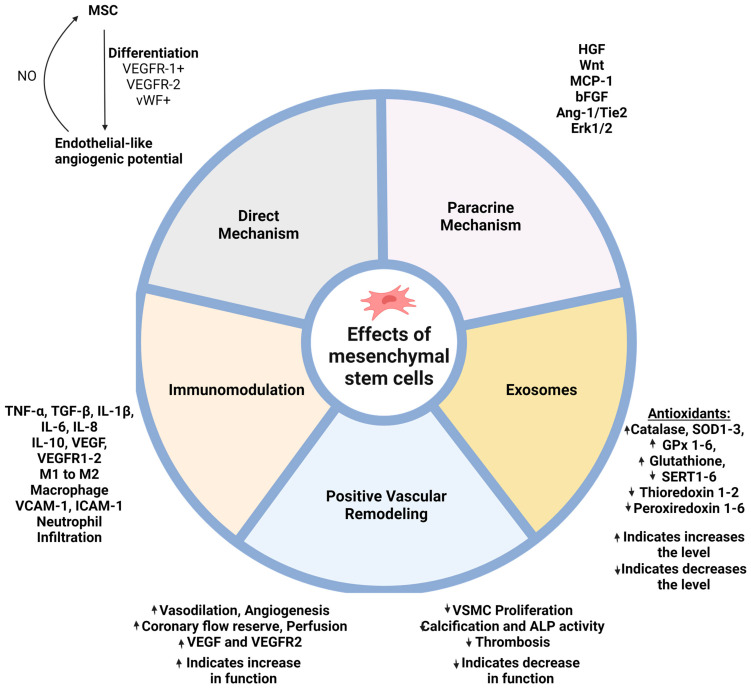
This figure summarizes the different ways MSCs can modulate the vascular modeling, which may contribute to making it a potential therapeutic approach enhancing AVF patency (made with biorender.com).

Figure 2 summarizes the different ways MSCs can modulate vascular modeling, which contributes to making it a potential therapeutic approach to enhance AVF patency. Inflammatory cytokines like MCP-1, IL1β, and TNF-α upregulation have been associated with various vascular diseases like atherosclerosis and pre-eclamptic hypertension mainly by intimal hyperplasia. Chemokines like MCP1 act as chemotactic agents, which allows for immune cell recruitment through CX3CR1/CCR2 and targeting these cytokines and chemokines, which are potential therapeutic strategies. Our group have reported that the adventitial delivery of adipose-derived MSCSs (AMSCs) improved vascular remodeling by significantly reducing the proinflammatory response with a higher shear wall stress and decreased the neointima/media ratio. Further, MSC-treated mice also showed a reduced M1/M2 ratio, supporting previous findings of targeting immune pathways. The multiple ways MSCs could possibly modulate vascular remodeling reinforce its application as a strong potential therapy [34,81,86,87,88].

There is evidence of a strong interaction of MSCs with ECs towards positive remodeling either by direct differentiation or MSC-EC cross talk through paracrine factors such as VEGF, VEGFR2, Ang-1/Tie2, MCP-1, IL-1β, fibroblast growth factors (bFGF), and matrix metalloprotease 2 (MMP2). An important player driving MSC-EC interaction is MSC-derived exosomes. Exosomes, also known as extracellular vesicles, contain a variety of RNA, proteins, and lipids with a diameter of 30–150 nm [89]. The exosomes derived from MSCs inhibited neointimal hyperplasia in a rat carotid artery injury model by activating the Erk1/2 signaling pathway and accelerating -reendothelialization [90]. The acceleration in reendothelialization is a strong indication to prevent thrombus and excessive neointimal formation. MSC-derived exosomes may also help in the modulation of inflammation, macrophage polarization, and secreting antifibrotic molecules [89].

## 5. Limitations and Considerations

While MSCs therapeutically have a lot of promising aspects, there are certain clinical and practical limitations that must be considered. Overall, using MSCs to treat AVFs is in its naïve research stage, so we need to think of a route of administration, frequency of treatment, affect over time, single dose vs. multiple doses, and autologous vs. allogenic sources. There are limited studies available in the literature on the adverse events of MSCs in all populations receiving MSC therapy. A review by Wang et al. summarized from a pooled analysis of 62 randomized clinical trials of MSCs therapy over 20 different disease types that the MSCs administrated was associated with 17 adverse events during the course of the MSCs therapy. Out of all of these, no serious safety events other than transient fever, administration site adverse events, sleeplessness, and constipation were discovered [91].

The financial implications of managing AVF stenosis warrant careful consideration. The current interventional treatments for maintaining AVF patency, such as angioplasty and stent placement, constitute a significant portion of annual expenditures for end-stage renal disease [45,92]. MSC-based therapies, which may prevent AVF stenosis and failure, could represent a cost-effective alternative to these interventional treatments [93]. However, MSC-based therapies present their own financial challenges. One major aspect is the substantial cost associated with ex vivo culture required to establish GMP (Good Manufacturing Practice)-grade stem cell colonies [94]. Autologous intra-arterial MSC delivery has proven to be highly expensive, as demonstrated in a study investigating the treatment of critical limb ischemia in type 2 diabetes mellitus [95]. The same research revealed that allogeneic MSCs, produced through mass manufacturing and administered intramuscularly, represent a more economically viable alternative [95]. Ongoing research efforts may identify additional strategies to enhance the cost-effectiveness and accessibility of MSC therapy for eligible patient populations.

## 6. Future Directions

While the potential of MSCs in enhancing arteriovenous fistula (AVF) patency is promising, several areas require further investigation as this approach translates into clinical practice. Future studies should focus on optimizing MSC delivery and determining the ideal timing, dosage, and route of administration for maximal efficacy in preventing AVF stenosis. Additionally, extended follow-up studies are crucial to assess the long-term safety and efficacy of MSC therapy in AVF patients, monitoring for potential adverse effects and evaluating the durability of the treatment’s benefits. Investigation into patient-specific factors that influence MSC therapy outcomes could lead to more tailored treatment strategies, potentially improving overall success rates. Comparative studies between different sources of MSCs (e.g., adipose-derived vs. bone marrow-derived) and between MSCs and other cell-based therapies are necessary to identify the most effective approach for AVF patency enhancement. Exploring the synergistic effects of MSCs with other therapeutic modalities, such as drug-eluting balloons or novel pharmacological agents, may yield more robust treatment options for AVF stenosis. The development of standardized protocols for MSC isolation, expansion, and quality control is crucial for consistent clinical application and regulatory approval. Research into MSC-derived products, such as exosomes or conditioned media, could potentially overcome some of the challenges associated with cell-based therapies while maintaining therapeutic efficacy. The identification of reliable biomarkers to predict and monitor patient response to MSC therapy could facilitate better patient selection and treatment optimization. Comprehensive cost-effectiveness studies are needed to evaluate the economic impact of MSC therapy compared to current standard treatments for AVF stenosis. Finally, the path forward involves a multidisciplinary approach, combining advances in stem cell biology, bioengineering, clinical medicine, and health economics to fully realize the potential of this innovative therapeutic strategy.

## 7. Conclusions

There is now significant work being carried out to understand the potential of MSCs in positive vascular remodeling by well-defined mechanisms. MSCs can modulate inflammation, cell-to-cell contact, and paracrine factors and impact several signaling pathways, making it a strong therapeutic agent to treat and have scope in enhancing AVF patency. While the initial preclinical/clinical studies have elaborated on the pathophysiology of MSCs and how safe they are in terms of their clinical perspectives, more studies need to be conducted to take this potential therapy from the bench to bedside to improve AVF failure in CKD patients undergoing hemodialysis.
